# A Trainable Open-Source Machine Learning Accelerometer Activity Recognition Toolbox: Deep Learning Approach

**DOI:** 10.2196/42337

**Published:** 2023-06-08

**Authors:** Fluri Wieland, Claudio Nigg

**Affiliations:** 1 Department of Health Science Institute of Sports Science University of Bern Bern Switzerland

**Keywords:** activity classification, deep learning, accelerometry, open source, activity recognition, machine learning, activity recorder, digital health application, smartphone app, deep learning algorithm, sensor device

## Abstract

**Background:**

The accuracy of movement determination software in current activity trackers is insufficient for scientific applications, which are also not open-source.

**Objective:**

To address this issue, we developed an accurate, trainable, and open-source smartphone-based activity-tracking toolbox that consists of an Android app (*HumanActivityRecorder*) and 2 different deep learning algorithms that can be adapted to new behaviors.

**Methods:**

We employed a semisupervised deep learning approach to identify the different classes of activity based on accelerometry and gyroscope data, using both our own data and open competition data.

**Results:**

Our approach is robust against variation in sampling rate and sensor dimensional input and achieved an accuracy of around 87% in classifying 6 different behaviors on both our own recorded data and the MotionSense data. However, if the dimension-adaptive neural architecture model is tested on our own data, the accuracy drops to 26%, which demonstrates the superiority of our algorithm, which performs at 63% on the MotionSense data used to train the dimension-adaptive neural architecture model.

**Conclusions:**

*HumanActivityRecorder* is a versatile, retrainable, open-source, and accurate toolbox that is continually tested on new data. This enables researchers to adapt to the behavior being measured and achieve repeatability in scientific studies.

## Introduction

### Background

The last decade has seen a significant increase in worldwide smartphone ownership [1], with approximately half of the world’s population now owning a smartphone and a device penetration rate of 80% in Germany and the United Kingdom [2]. Even low-end smartphones are equipped with various sensors, including accelerometers, gyroscopes, proximity sensors, magnetometers, and GPS receivers, along with energy-efficient processors and stable internet connections. With the advent of smartphones and wearables, physical activity analysis has greatly gained in popularity. Accelerometry-based behavior analysis has a variety of applications, such as fall detection in older patients [3], health monitoring [4], work-related stress analysis [5], and sleep analysis [6]. The widespread use of accelerometry in everyday smartphone apps has reduced the cost of gyroscope and accelerometer sensors, which has in turn accelerated their development. While wearables have gained popularity as accelerometer devices, smartphones still make up the majority of them.

Many studies have shown the accuracy and reliability of smartphone sensors in accelerometry [7-9]. Although wearables tend to provide more accurate behavior classifications, the potential of using smartphones far outweighs the additional accuracy gained from wearables. Although they are more precise thus far [10], the cost of wearables for larger study populations is very high, compared with the widespread popularity and affordability of smartphones, making them a more accessible option for research. Additionally, smartphone apps are easier to distribute, update, configure, and adapt to specific research questions than wearables. Wearables also have the disadvantage of limited software support and closed-source software, making research based on previous software nonreproducible after algorithm updates. This means that wearables bought for research purposes must be replaced on a regular basis.

Most importantly, however, the default software of wearable manufacturers is in almost all cases not open-source, meaning that after each change of the algorithm (ie, app update) that classifies behavior, research based on previous software is not reproducible anymore. Furthermore, in most cases, charges apply for the use of the said software. On the other hand, some smartphone manufacturers offer free, open-source toolboxes for movement activity recognition, such as Samsung and Huawei. However, these toolboxes only recognize a limited number of activity types and are at the time of writing not trainable to new activities. The purpose of both, however, is for them to be integrated into applications, so they can be used to determine whether a smartphone user is moving and is active or not, in order to interact with application functionality, such as energy saving while not moving, clocking active hours, or encouraging movement when a user is inactive. While data can be collected and stored, the behavior classes are fixed and neither trainable nor retrainable. To address these limitations, the scientific community needs access to an open-source, adaptable behavior analysis toolbox that also facilitates reproducible research and is adaptable to specific research questions. To fulfil this need, we present our open-source, deep learning–based behavior analysis toolbox. Our Human Activity Analysis toolbox includes a proprietary Android app, 2 deep learning algorithms, scripts to process data, and a continually expanding sample data set. The toolbox has been validated with a sample of 68 University of Bern students and employees.

### Activity Recognition and Deep Learning Background

Deep learning algorithms have gained importance in classifying human behavior based on sensor data collected from accelerometers, gyroscopes, and magnetometers [11-18] (for a deeper understanding and comprehensive overview, see [19]). These algorithms are based on artificial neural networks, and specifically, deep neural networks (DNNs) have become the dominant approach for activity recognition as of 2022. DNNs consist of multiple layers of neurons of similar or different types, and the functionality of these neurons is determined by the nature of the layers and the way they are interconnected [20,21]. It is important to note that a standard neural network consists of many simple, connected processors called neurons, each producing a sequence of real-valued activations. Depending on the problem and how the neurons are connected, such behavior may require long causal chains of computational stages. Thus, if multiple layers of neurons are used sequentially, we speak of DNNs [20].

Most DNN architectures consist of a convolutional neural network (CNN) layer, followed by either a feedforward neural network (FNN) layer or a recurrent neural network (RNN) layer. Unlike the output from an RNN neuron, which is fed back into the same layer, the output from an FNN neuron is only connected to the next layer. CNNs handle variable input dimensions quite well and are mainly used for feature extraction for the RNN or FNN layer, which, combined with a prior CNN, output a better generalization than if fed with raw sensor data [22]. However, FNNs only work well with data of the same input dimensions, and RNNs only work with a fixed number of streams. As a result, the widely used CNN-RNN-FNN combinations do not work with varying input dimensions. This means that if data collection from one sensor stops, the movement type cannot be classified by the DNN that was trained on multiple input dimensions. In order to save battery life in smartphones during long-term recordings, it is often desirable to temporarily disable certain sensors or to vary the sampling rate of sensors, which results in changing the input dimensions for the DNN.

When a participant is sitting for an extended period, disabling the gyroscope sensor can conserve battery life. This is because the rotational position is unlikely to change significantly without significant acceleration changes unless the person is in an aircraft and the gravitational acceleration is being compensated for in the data. In order to determine when the activity type changes, it is sufficient to use a low recording frequency. This means that it is possible to deactivate the gyroscope and magnetometer and lower the accelerometer recording frequency. To determine when the activity type changes, a very low recording frequency suffices, so it is desirable to deactivate the gyroscope and magnetometer and lower the accelerometer recording frequency significantly. Dummy data can be generated to compensate for missing data in order to maintain the accuracy of the trained CNN-FNN-RNN model [23]. However, this approach can result in a loss of accuracy in classification. Another solution is to insert a global pooling layer, but this also leads to a reduction in accuracy. This, however, leads to accuracy loss in classification. Another solution is to insert a global pooling layer [24], but this also leads to a reduction in accuracy.

Previous publications on accelerometry-based movement recognition have shown great success but significant limitations. Ordóñez and Roggen [15] presented a deep-CNN–based framework, which they tested against models such as decision tree, random forest, and support vector machines. Trained and then tested on a data set, the accuracy reached up to 86.7%. The authors then analyzed which component of the data had the biggest impact on classification accuracy and determined this to be changes in acceleration, which is in line with our own results.

Wang et al [11] offer a comprehensive survey of recent advancements in activity recognition and associated methodologies. Their work sheds light on the various strengths and weaknesses of deep learning models when it comes to activity classification. Although most models perform accurately on their trained data [25], significant limitations remain. First, the lack of extensive, labeled accelerometry data sets limits their efficacy. Second, the generalization capabilities of models need improvement. Third, models struggle with sensor noise and input variability, highlighting a need for greater robustness. Our algorithms aim to address these issues, working to mitigate the associated limitations and enhance overall model performance. To achieve this, we build upon previous research by incorporating and improving upon their methodologies while also introducing our own additional data set for algorithm training.

Malekzadeh et al [26] proposed a new model, which tries to counteract the aforementioned shortcomings by introducing a *dimension-adaptive pooling* (DAP) layer, which makes DNNs robust to changes in not only sampling rates but also dimensional changes of the data due to varying sensor availability.

The authors also introduced a *dimension-adaptive training* layer, and combined it with the classical CNN-FNN-RNN approach and the DAP layer. They claim that dimension-adaptive neural architecture (DANA) can prevent losses in classification accuracy, even under varying sensor availability and temporal sampling rate changes. This model was tested on 4 publicly available data sets, including the MotionSense [27] data set, which consists of accelerometer data from 24 students at Queen Mary University of London.

Our goal was to not only implement this model into our own DNN, but also to improve upon it and validate it using our own data. The robustness of the DANA model is very promising, making it a valuable addition to our research.

## Methods

### Ethical Considerations

According to the guidelines stated on the Ethics Commission page of the University of Bern's Faculty of Human Sciences, no ethics committee approval was required for this research. This conclusion is based on the fact that all data was collected with participants' informed consent, the data collection was conducted anonymously, and the research activities only involved non-hazardous tasks such as standing, sitting, walking, and ascending or descending stairs. No personal data was collected.

### Training Data

**Figure 1:**
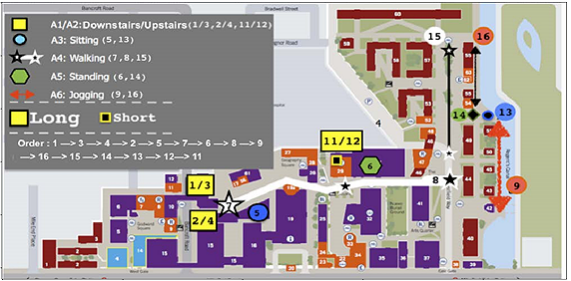
Course for accelerometer data collection on the campus of the Queen Mary University of London for the MotionSense data set; graph from Malekzadeh et al [26].

The data used for the initial training of the neural network was gathered from the MotionSense Github repository. These data consist of accelerometer and gyroscope readings from an iPhone 6s (Apple Inc), collected at a frequency of 50 Hz by 24 participants who followed a set of actions on the campus of Queen Mary University of London. These actions included ascending or descending stairs, sitting, walking, standing, and jogging (Figure 1). The data recorded gravity, acceleration, rotation, and attitude on 3 axes.

**Figure 2:**
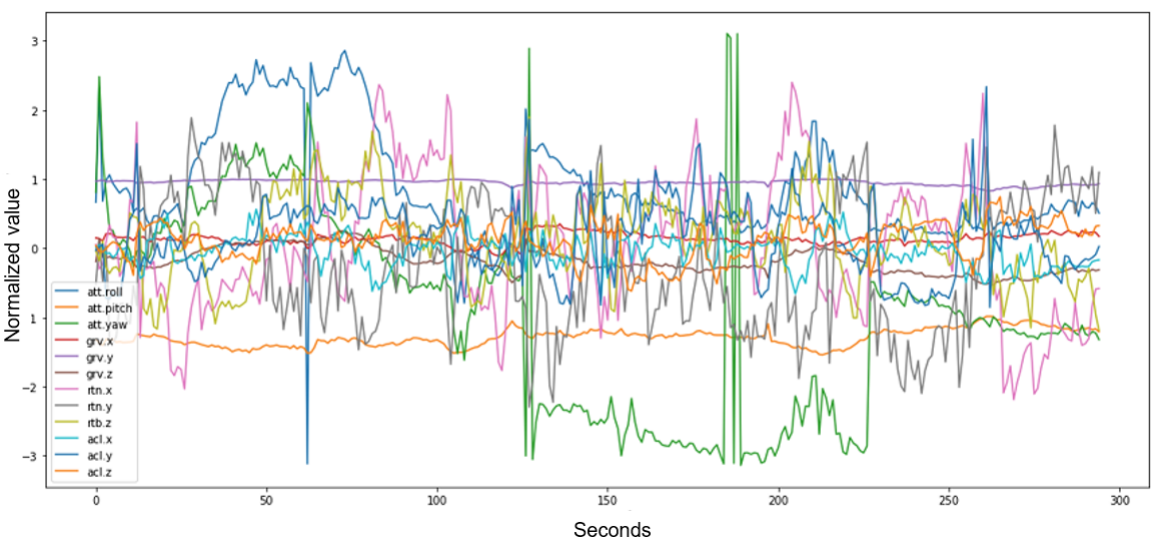
Data example of the MotionSense data set. Note that some values do not change significantly when normalized over the course of recording and are therefore of lesser interest for the prediction of behavior.

After conducting a principal component analysis, we found that the X, Y, and Z acceleration and rotational changes were the most predictive factors in classifying the participant’s behavior (Figure 2). Therefore, only these 6 values were used in the training of the algorithm. As a result, our app only records these 6 values, which are then used for further analysis.

To gather more data and validate our model, we set up our own course of action on the campus of the Centre for Sports Science at the University of Bern, modeled after the course used at Queen Mary University. A total of 68 participants (aged 21-59, median 26, SD 3.2 years), who were students and employees of the University of Bern, completed the course while our *HumanActivityRecorder* Android app (Multimedia Appendix 1) was running and collecting data. All participants were fully informed about the task and gave their consent for the data collection.

**Figure 3:**
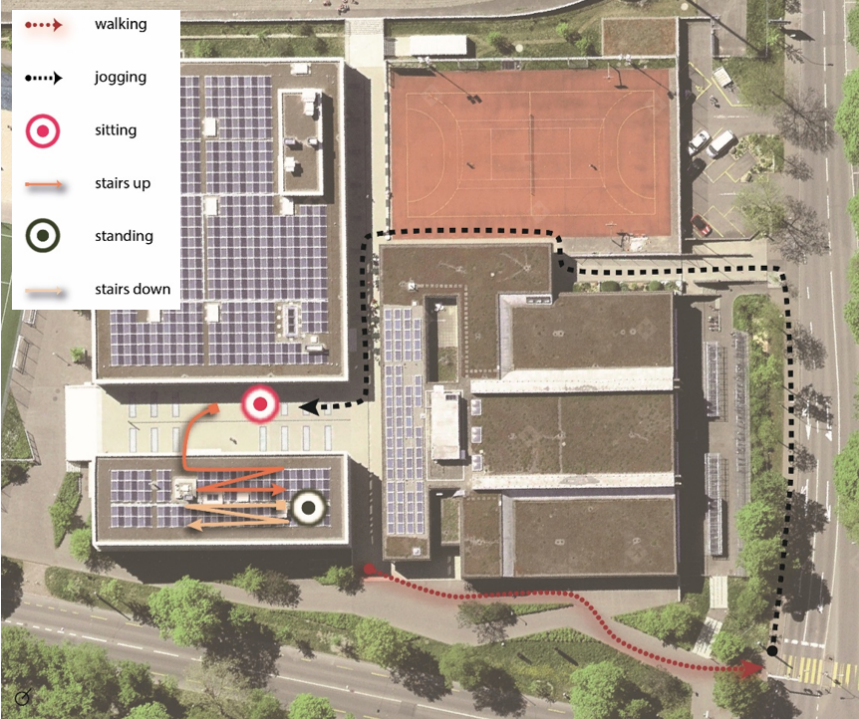
Course on the premises of the University of Bern. Participants followed the indicated path, starting walking, followed by jogging, sitting, ascending stairs, standing, and descending stairs. Completion took an average of approximately 300 seconds.

The course consisted of approximately 300 seconds of walking, jogging, sitting, and walking up and down stairs and standing still (Figure 3). All participants completed all segments of the course, and the corresponding data segments were manually labeled for use in training the models.

The participants completed the course in 2 groups with different instructions. Group 1 (n=29, median age 26, SD 5.2 years) was instructed to wear the smartphone in their preferred manner. Group 2 (n=39, median age 27, SD 4.7 years) wore the smartphone in the right front trousers’ pocket, with the display facing toward the body and the top of the phone pointing down while standing. This placement is consistent with the data collection method used for the *MotionSense* data set, as discussed above. It was found that the orientation of the smartphone has a significant impact on the performance of the model. To ensure consistency and comparability between the data sets, our algorithm was trained on the data of group 2, as wearing the smartphone in an individually preferred manner (group 1) resulted in significantly worse performance in classification accuracy. For a detailed comparison of classification accuracy between groups 1 and 2, please refer to Multimedia Appendix 2.

### App

**Figure 4:**
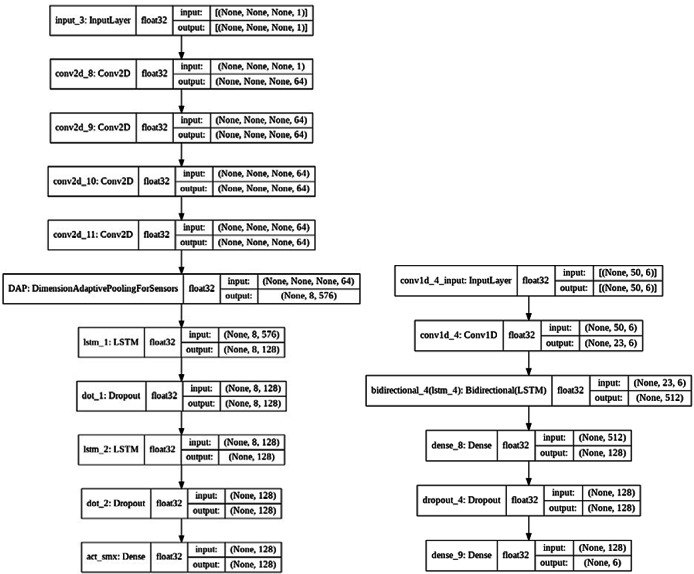
Comparison of the models used in our study. The dimension-adaptive neural architecture (DANA) model, consists of several additional layers, which we found did not improve the classification of our data. Note that in our simplified model, the dimension-adaptive pooling (DAP) layer has been omitted as well, since our data are dimensionally consistent. LSTM: Long short-term memory.

The accelerometer and gyroscope data were collected using our custom-made *HumanActivityRecorder* Android app, which was developed using Android Studio 4.1 with Java 1.8.0_271 (Figure 4). The app records accelerometer and gyroscope data at a sampling rate of 50 Hz and is publicly available on the Google Play Store as version 13 of the *HumanActivityRecorder* app. The accelerometer data are recorded in the x-, y-, and z-axes, while the gyroscope data consist of rotation around these axes (roll, pitch, and yaw) at the same frequency. The data are then automatically sent to a server and can be downloaded as a CSV or JSON file. The source code is available on Github [28]. The app is compatible with Android 5.0 and later versions. We used an Honor View 20 smartphone for data collection to ensure consistency in recording. Only 1 device was used.

### Recording

Before beginning the data collection process, the participants were asked for their name, age, and consent. The data collection paradigm was explained to them and demonstrated through a walk-through by the data collector. The participants then completed the course, which included walking, jogging, sitting, ascending and descending stairs, and standing still, while the app recorded their accelerometer and gyroscope data. After completing the course, the participants were given a chocolate bar as an incentive. The accelerometer data were processed and categorized using a Jupyter notebook script, which automates the workflow to ensure consistency in categorization. This script is part of our toolbox.

### Deep Learning Model

We implemented a modified version of the DANA model proposed by Malekzadeh et al [19], which involved removing and modifying several layers. This modification was made after testing the model (trained and tested on *MotionSense* data) and finding that the omission of these layers did not noticeably decrease the model’s performance.

It is important to note that in our simplified model, we removed the DAP layer as our input data are dimensionally consistent at the time of testing. To validate the models, we trained them both on the *MotionSense* data set and our own data set, as well as testing both combinations.

## Results

Through a systematic variation of the number of nodes and layers, we determined that the best balance between accuracy and complexity is achieved with the described architecture. This architecture was determined based on the accuracy of the models in classifying movement types of the *MotionSense* data set when trained on the same data set. Interestingly, when we trained on the *MotionSense* data set and tested on our own data, our model performed better than DANA, yet still with room for improvement, at 63% vs 26%.

**Figure 5:**
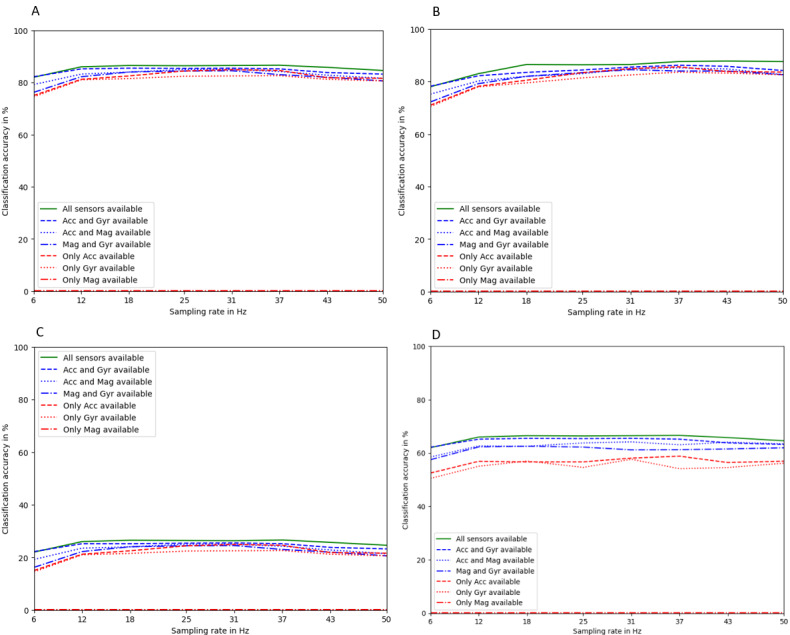
Accuracy in classifying using the dimension-adaptive neural architecture (DANA) model (A) trained and tested on MotionSense data; (B) our model trained and tested on our data; (C) DANA trained on MotionSense and tested on our data; and (D) our model trained on our own data and tested on MotionSense data. Note that the dimensionality is varied here to showcase the robustness, and our model is impacted more strongly by a varied dimensionality input. Acc: accelerometer; Gyr: gyroscope; Mag: magnetometer.

**Figure 6:**
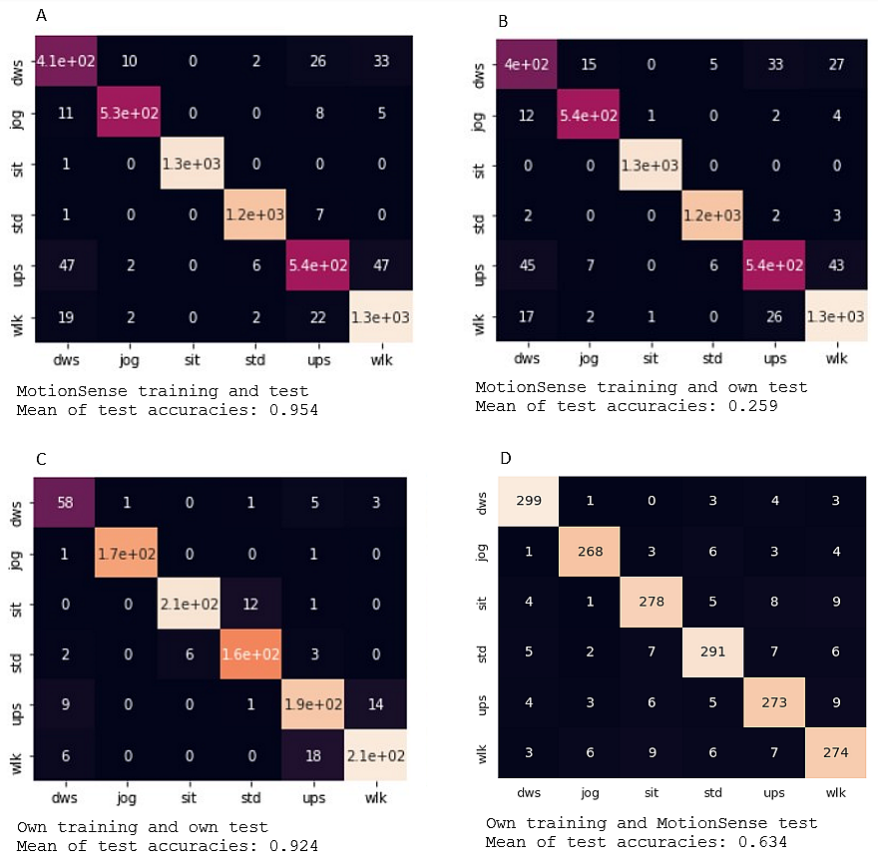
Confusion matrices of accuracy in classifying (A) using our own simplified model trained on MotionSense data tested on MotionSense data; (B) trained on MotionSense data and tested on own data; (C) trained and tested on our own data; and (D) trained on our own data and tested on MotionSense data. Note that dimensionality is not varied here as all sensors are available. dws: downstairs; jog: jogging; sit: sitting: std: standing; ups: upstairs; wlk: walking.

When trained on the same data set as the one they are tested on, both models performed well in classifying behavior. The DANA model achieved approximately 87% accuracy when trained and tested on the MotionSense data set and approximately 90% accuracy when trained and tested on our own data, depending on the sampling rate (Figure 5). However, when trained on the MotionSense data set and tested on our own data, the accuracy of DANA drops to around 26%, also depending on the dimensionality of the input, while our model performs at around 63%, but much less robust against the dimensionality input (Figure 6). This still leaves room for improvement but shows the comparatively high generalization ability of our model. It is important to note that neither the MotionSense data nor our own data include magnetometer data, which is why the DANA model performs poorly (at or near zero accuracy) when reduced to only magnetometer input. The graph includes this information for consistency.

Our simplified model does not include the DAP layer and is less robust against input dimensional variance, as our input data dimensions did not vary. However, it is easily adaptable if desired. Despite this, our model outperforms the DANA model in terms of accuracy. When trained on the MotionSense data set and tested on it, our model achieved 95.4% accuracy. It was equally accurate when trained on our own data and tested on it, with 92.4% accuracy. However, when trained on the MotionSense data and tested on our own data, accuracy drops to 25.8%, but when trained on our data and tested on MotionSense, accuracy reached 63.4%.

## Discussion

### Conclusions

Both models included in our toolbox perform well when trained and tested on the same data set. However, they do not perform well when trained on one data set and tested on the other, as was the case in our study. This highlights the problem of the unavoidable part of overfitting the collected data to improve algorithm performance, although this is controlled for as far as possible. Despite this, both models (DANA and our own) performed similarly when trained on one data set and tested on the other. Our model is slightly more accurate, but the DANA model is more robust with regards to dimensional variance in the input. However, there is a significant difference in computing time when training the models. The DANA model, when trained using Google Colab with CPU and GPU resources, took around 11 hours to train each time. On the other hand, our model can be trained in about 5 minutes with 100 epochs of training using only CPUs in Google Colab. Note that this estimation does not include hyperparameter testing.

Given the amount of data used to train the models, the results are surprisingly accurate. Commercial wearables, such as sports-oriented smartwatches, often have a function to display the user’s current activity. However, these displayed activities are often incorrect, even for activities that seem obvious to the user. Considering these devices are widely available and sold to millions of people, we expected movement detection to be much more challenging, and our accuracy to be in the low 60% range.

While the accuracy of movement classification is very good, there is still room for improvement, which we plan to achieve by training the algorithm on additional data from diverse populations or environments. We recommend using the DANA model to classify behavior in data that have been gathered at different dimensions or with variable input dimensions. However, if the input type is consistent, we recommend our model as it is slightly more accurate and much easier to train. Both algorithms are available at our Github repository, along with the *HumanActivityRecorder* app and the scripts to process the data. In a future step, we plan to integrate both algorithms into the app and evaluate their performance in a subsequent study.

### Limitations

The orientation of the smartphone during recording has an impact on classification accuracy if the sample size is not large enough, as shown in our comparison of classification accuracy of groups 1 and 2 (Multimedia Appendix 2). However, if trained on large data sets with varying orientation, this effect disappears. For comparability, we based our model on the group with the same orientation as in the *MotionSense* data set. Accounting for orientation was outside the scope of our study. To address the impact of smartphone orientation on classification accuracy in medium-sized samples, an easy solution would be to incorporate an orientation recognition stage that detects the orientation of the smartphone and branches the data to models that have been individually trained on each orientation. This would ensure more accurate classification regardless of the smartphone orientation.

### Authenticity

The results of the study are presented clearly, honestly, and without fabrication, falsification, or inappropriate data manipulation. The results of this study do not constitute endorsement by this Journal. This manuscript has not been published elsewhere, and it has not been submitted simultaneously for publication elsewhere.

## Appendix

Multimedia Appendix 1 Screenshots of the Android app. From left to right: start screen, sociodemographics, and recording screen.

Multimedia Appendix 2 Accuracy of the classification of our model (A) trained and tested on group 1 data; (B) trained on group 1 data and tested on MotionSense data; (C) trained and tested on group 2 data; and (D) trained on group 2 data and tested on MotionSense data. Group 1 was instructed to wear the smartphone wherever they preferred individually. Group 2 was instructed to wear it screen inside, top facing downward in the right trouser pocket, in line with data collection for the MotionSense data set, to ensure maximum comparability.

## Abbreviations

CNN: convolutional neural network
DANA: dimension-adaptive neural architecture
DAP: dimension-adaptive pooling
DNN: deep neural network
FNN: feedforward neural network
RNN: recurrent neural network

## Data Availability

All data used are available [28].
