# Supplementary figures and images for: A Trainable Open-Source Machine Learning Accelerometer Activity Recognition Toolbox: Deep Learning Approach
Source: JMIR AI. 2023 Jun 8;2:e42337. doi: 10.2196/42337 (PMC11041400; doi:10.2196/42337)

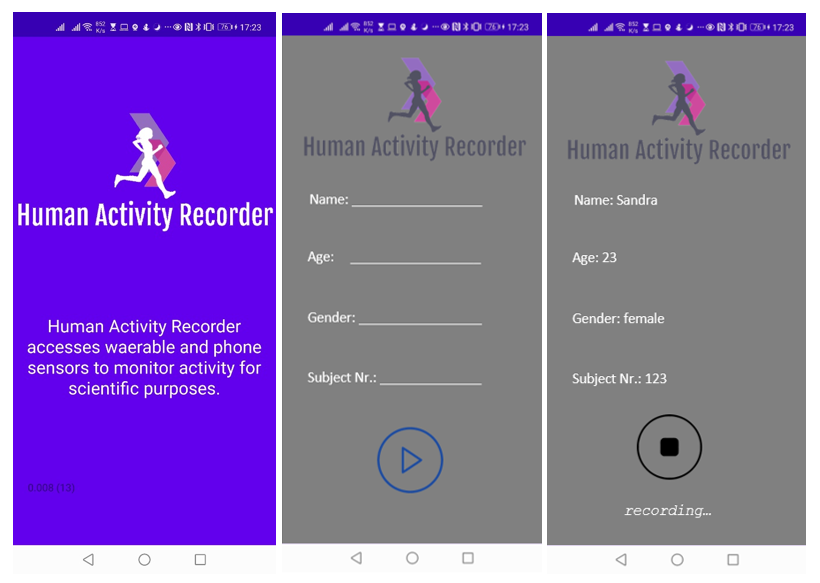

Supplement: Multimedia Appendix 1 [file ai_v2i1e42337_app1.png]

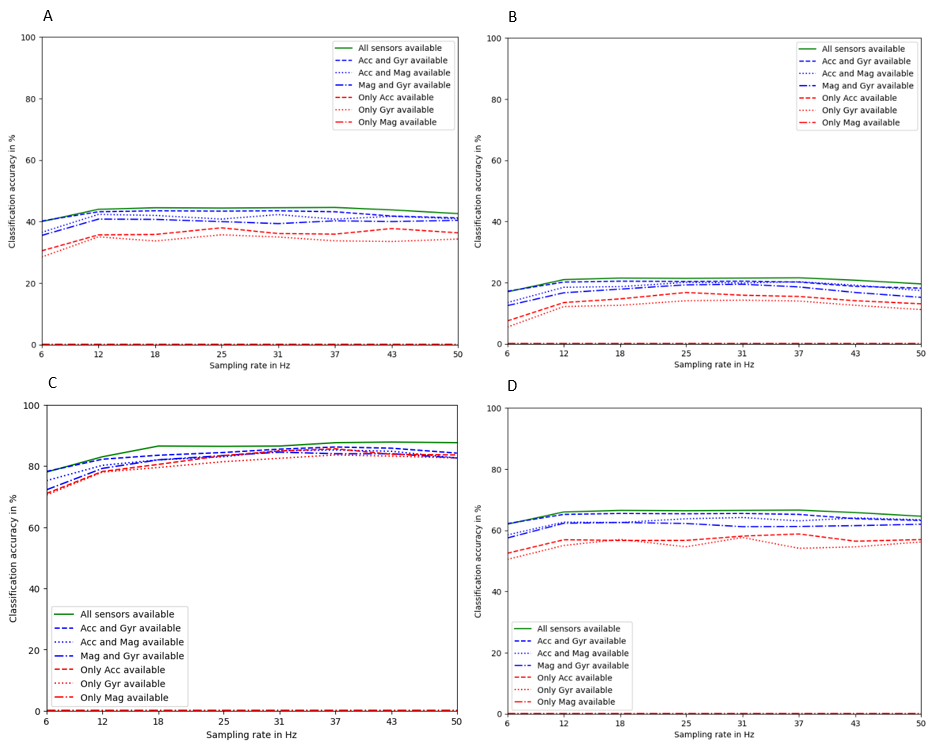

Supplement: Multimedia Appendix 2 [file ai_v2i1e42337_app2.png]
